# Human–Computer Interaction-Oriented African Literature and African Philosophy Appreciation

**DOI:** 10.3389/fpsyg.2021.808414

**Published:** 2022-01-07

**Authors:** Jianlan Wen, Yuming Piao

**Affiliations:** ^1^School of Foreign Languages, Yanbian University, Yanji, China; ^2^School of Foreign Languages, Jilin Institute of Chemical Technology, Jilin, China

**Keywords:** African literature, African philosophy, HCI, HMD-VR, CNN, EEG

## Abstract

African literature has played a major role in changing and shaping perceptions about African people and their way of life for the longest time. Unlike western cultures that are associated with advanced forms of writing, African literature is oral in nature, meaning it has to be recited and even performed. Although Africa has an old tribal culture, African philosophy is a new and strange idea among us. Although the problem of “universality” of African philosophy actually refers to the question of whether Africa has heckling of philosophy in the Western sense, obviously, the philosophy bred by Africa’s native culture must be acknowledged. Therefore, the human–computer interaction-oriented (HCI-oriented) method is proposed to appreciate African literature and African philosophy. To begin with, a physical object of tablet-aid is designed, and a depth camera is used to track the user’s hand and tablet-aid and then map them to the virtual scene, respectively. Then, a tactile redirection method is proposed to meet the user’s requirement of tactile consistency in head-mounted display virtual reality environment. Finally, electroencephalogram (EEG) emotion recognition, based on multiscale convolution kernel convolutional neural networks, is proposed to appreciate the reflection of African philosophy in African literature. The experimental results show that the proposed method has a strong immersion and a good interactive experience in navigation, selection, and manipulation. The proposed HCI method is not only easy to use, but also improves the interaction efficiency and accuracy during appreciation. In addition, the simulation of EEG emotion recognition reveals that the accuracy of emotion classification in 33-channel is 90.63%, almost close to the accuracy of the whole channel, and the proposed algorithm outperforms three baselines with respect to classification accuracy.

## Introduction

Fifty-four nations make up Africa, and each of these countries has its own history, culture, tribes, and traditions. There are four types of African literature, which are oral literature, pre-colonial African literature, colonial African literature, and post-colonial African literature (Mphande, 2020). The characteristics of African literature include slave narratives, protest against colonization, and calls for independence. African literature is classified as traditional oral literature, literature written in indigenous African language, and written in European languages (Van Oostrum, 2020; Eatough, 2021).

Africa is a huge place comprising many countries, cultures, and languages. Therefore, claiming that a single philosophical tradition or culture pervades the continent is an oversimplification of the realty, even those who argue that a uniting Bantu Philosophy is only focusing on areas with the shared Bantu language and cultural tradition (Aliye, 2020). Confusingly, does African philosophy exist, and if so, in what form? The majority, though not all, of ancient African philosophy is oral (Presbey, 2020). Therefore, it is more difficult to nail down the meanings of particular terms, concepts, or positions. There is also very little comparatively written about African philosophy out there compared to even East Asian philosophy, Islamic philosophy, traditional Western European philosophies. Due to this, some scholars try to construct some ethno philosophy and infer the kind of philosophy from culture.

Philosophy and literature strive to make a contribution to humanities by bringing together two fields that are independently complex. The main heuristic for the development of African literature and African philosophy is the increasing literacy rate. African literature has its unique style and connotation, and its emergence has such a short but rich development course. Rich tradition, borrowing from the West, and facing reality are the characteristics of African literature. African literature has themes of liberation and racial difference, and the sensitivities and ideologies of the writers have determined their role in the course of history. African literature has exquisite art forms, but more importantly, it conveys profound and complex ideas. Almost all African writers will integrate certain philosophical thoughts into their works.

Virtual reality (VR) is a three-dimensional virtual environment generated by computer simulation. By using human–computer interface (HCI) technology. VR can provide users with multichannel sensory simulation such as vision, hearing, touch, taste, smell, and so on, which introduces a highly immersive experience to users (Xiao et al., 2019). Compared with traditional computer technology, VR can provide a more natural way of HCI, emphasizing the dominant role of human in the interaction. VR systems are characterized by interaction, immersion, and imagination. As an observation device, head-mounted display (HMD) can perfectly meet the characteristics of enjoying the African literature (Zhu et al., 2019).

Given the above, through the HMD-VR environment the reflection of African philosophy in African literature can be enjoyed. The electroencephalogram (EEG) is used to study emotion recognition, and the data is transmitted to the control center to capture the user’s psychological state on the analysis of multiscale convolution kernel convolutional neural networks (CNN), pushing more suitable and more wonderful scenes for user’s emotional fluctuations (Zavala-Mondragon et al., 2020; Gao Q. et al., 2021).

Accordingly, the main contributions of this work are summarized as follows. (i) A physical object of tablet-aid is designed, and depth camera is used to track the user’s hand and tablet-aid and map them to the virtual scene, respectively. (ii) A tactile redirection method is proposed to meet the user’s requirement of tactile consistency in virtual space and real space. (iii) EEG emotion recognition based on multiscale convolution kernel CNN is proposed to appreciate African literature and African philosophy.

The rest of this work is organized as follows. Section “Related Work” reviews the related work. In Section “Human–Computer Interaction in Head-Mounted Display-Virtual Reality Environment Based on Position Tracking and Tactile Redirection,” the HCI in HMD-VR environment based on position tracking and tactile redirection is studied. In Section “Electroencephalogram Emotion Recognition Based on Multiscale Convolution Kernel Convolutional Neural Networks,” EEG emotion recognition based on multiscale convolution kernel CNN is proposed. The experimental results are shown in Section “Experiment and Results Analysis”. Section “Conclusion” concludes this paper.

## Related Work

Virtual reality environment is a three-dimensional space environment similar to the real world, and its interaction modes include navigation, selection, and manipulation. Navigation refers to taking users from one place to another in a virtual environment. Selection allows the user to select one or more objects in the virtual environment. Manipulation usually refers to modifying the position and direction of an object. There are many studies on interaction problem in HMD-VR environment. Kang (2020) proposed a research model to analyze the influence of tourism product for HMD-VR through emotional and cognitive processes. HMD-VR also played an important role in education, and in Valentine et al. (2021), the colleges evaluated the benefits of HMD-VR in implementation without manipulating the device from students. HMD-VR provided a unique chance for sports by understanding the changing of sensing environment, and in Anglin et al. (2017), the authors studied whether the adaptation of visual motion rotation task in HMD-VR had a similar adaptation effect in traditional training. HMD-VR could also be used as a supplementary to medical treatment, and in Austin et al. (2021), the authors found that HMD-VR could effectively relieve neuropathic pain in patients with spinal cord injury. Also, in Rose et al. (2021), authors explored the feasibility of HMD-VR in inpatient psychiatric nursing environment. Meanwhile, the produced movements in VR provided hopes for rehabilitation, and in Wang et al. (2020), the effect of HMD-VR on neural data integrity was investigated.

Emotion recognition has always been the challenging problem in computer visual field. Some strategies have been proposed to recognize emotion through neural network. In Jain et al. (2019), a facial emotion recognition method was proposed, which was based on single deep convolutional neural network. In Song et al. (2020), a dynamic graph-based CNN was proposed for multichannel EEG emotion recognition. In Shahin et al. (2019), a Gaussian hybrid model and a deep neural network-based hybrid classifier emotion recognition method was proposed to realize the text- and speaker-independent emotion recognition system. In Yu (2021), a deep neural network-based human emotion recognition method was presented to improve human emotional communication and learning behavior. In Wang et al. (2019), authors proposed a multichannel EEG emotion recognition method based on phase locked value map CNN. In Lee et al. (2020), a voice emotion recognition-based communication system was proposed to preprocess voice through voice data enhancement method, and then, CNN was used to recognize five emotions. In Shen et al. (2020), a four-dimensional convolutional recurrent neural network-based emotion recognition method was proposed to improve the accuracy of emotion recognition based on EEG. In Batbaatar et al. (2019), a semantic emotion neural network was presented for text emotion detection. In Gao Z. et al. (2021), a multilayer CNN combined with differential entropy and brain network-based emotion classification method was proposed. In Zheng et al. (2021), a three-dimensional feature map and CNN-based emotion recognition method was proposed to improve the accuracy in emotion recognition based on EEG. To sum up, there are few works on immersion and cybersickness, and so this work mainly focuses on improving some problems in HMD-VR. Unlike previous works, this work uses HCI-oriented method to appreciate African literature and African philosophy creatively.

## Human–Computer Interaction in Head-Mounted Display-Virtual Reality Environment Based on Position Tracking and Tactile Redirection

With the evolution of technology into daily life, we now live in a world of experience driven global connectivity. People share moments on social media, but today it is unlocking the power to help us tell stories better than we have ever before, and with the rise of VR and augmented reality (AR), technologies are being given a new opportunity to bridge those communication gaps even further through direct experience. In the HMD-VR environment, African philosophical ideas are introduced into African literature through HCI system based on tablet-aid, which is presented intuitively to users in VR to improve users’ immersion. Smart tablet is a very familiar interactive device in our live, which has a mature interactive paradigm using touch screen. Therefore, this work hopes to apply the interactive paradigm of tablet device to HMD environment. To solve the comprehensive interaction problem in HMD-VR environment, it is necessary to see and use tablet device intuitively and efficiently in an HMD environment.

### System Design

**FIGURE 1:**
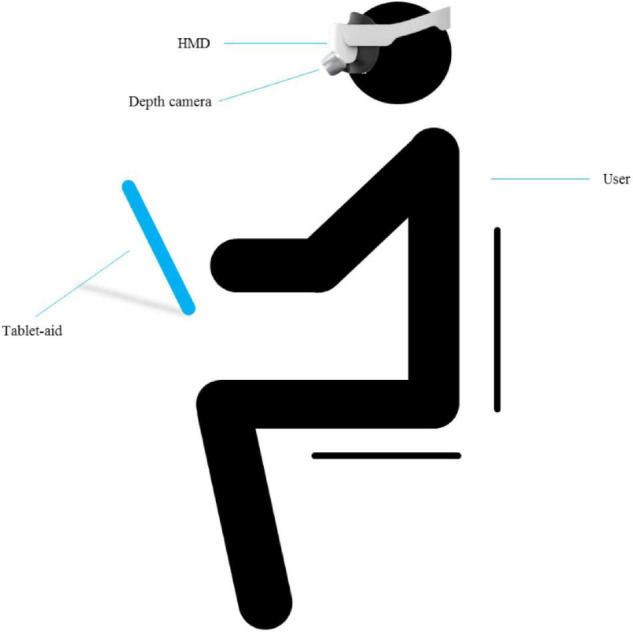
The diagram of HMD-VR interactive system.

The HMD-VR interactive system designed in this work introduces the interaction paradigm of smart tablet into the HMD environment by using tablet-aid, which not only conforms to people’s usage habits, but also provides users with tactile feedback consistent with virtual and real. The system design is shown in Figure 1. The depth camera is mounted in front of the HMD and tilted down about 15°–20° to eliminate visual differences due to the position difference between the depth camera and the user’s eye. Depth camera is the eye of the terminal and is also called 3D camera, which can detect the depth distance of the shooting space through the camera.

In the HMD-VR environment, users cannot see hands and tablet-aid in the physical scene, which makes it difficult to conduct accurate position interaction. Therefore, depth camera is used to track tablet-aid *ta* (the position of *ta* is *P*_*ta*_) and user’s right hand *rh* (the position of *rh* is *P*_*rh*_). This work assumes that the user is dextromanuality. The user picks up *ta* with left hand and interacts with the button on the virtual tablet with right hand, which are mapped to the virtual scene into the virtual hand *vh* (the position of *vh* is *P*_*vh*_) and the virtual plane *vp* (the position of *vp* is *P*_*vp*_), respectively.

The problem of introducing smart tablet interaction paradigm into HMD-VR environment lies in the consistency of virtual and real. Therefore, this work adopts position tracking and tactile redirection technologies to achieve the related goal, and makes improvements and optimizations according to the requirements.

### Position Tracking

Leap motion controller has been around here for quite some years now, and it has been used by so many people regarding VR. The leap motion controller allows programs to be controlled with natural gestures, freeing them from traditional mouse and keyboard controls. Leap motion is commonly used to restore hand posture and get hand position due to its lowly interaction and immersion.

In the depth camera coordinate system is located the *ta*, and its position coordinate is *P*_*ta*_(*a*, *b*, *c*). Also, *vp* is located in the world coordinate system in virtual scene, and its position coordinate is *P*_*vp*_(*a*_0_, *b*_0_, *c*_0_). Since *ta* and *vp* are in different coordinate systems, it is necessary to transform *ta* into the world coordinate system through the coordinate system transformation matrix ***A***, and then eliminate the position difference between *ta* and *vp* in the same coordinate system through the transition matrix ***A*_1_** to synchronize the position of *ta* and *vp*. Among them,


$$A=\left[\begin{array}{cccc} 1 & 0 & 0 & 0\\ 0 & 1 & 0 & 0\\ 0 & 0 & 1 & 0\\ a_{0} & b_{0} & c_{0} & 1 \end{array}\right]$$

$$A_{1}=\left[\begin{array}{cccc} 1 & 0 & 0 & 0\\ 0 & 1 & 0 & 0\\ 0 & 0 & 1 & 0\\ T_{a} & T_{b} & T_{c} & 1 \end{array}\right]$$

The space mapping process is defined as follows.


(1) $$P_{vt}^{\prime }=P_{ta}^{\prime }AA_{1}$$

where $P_{vt}^{\prime }$ and $P_{ta}^{\prime }$ are the homogeneous coordinate description of *P*_*vp*_ and *P*_*ta*_, respectively. The position *P*_*rh*_ can be traced through the depth camera. Since *rh* and *vp* are in different coordinate systems, the coordinate system transformation matrix ***A***’ and transition matrix ***A*_1_** can be similarly transformed as follows.


(2) $$P_{vt}^{\prime }=P_{rh}^{\prime }A^{\prime }A_{1}$$

where $P_{vt}^{\prime }$ and $P_{rh}^{\prime }$ are the homogeneous coordinate description of *P*_*vp*_ and *P*_*rh*_. According to Eqs 1, 2, *ta* and *rh* can be in the same interactive space with *vp* through coordinate transformation.

### Tactile Redirection

The only interaction mode of smart tablet is hand contact, and the high unity of touch and visual effects is the guarantee of efficient interaction. In this system, the object contacts by user is the tablet-aid *ta*, and its mapping object is the virtual tablet *vp*. In HMD-VR environment, the user’s intuitive operation target is *vp*. Therefore, it is not expected that the *vp* size is too small in virtual world to affect the efficiency and accuracy of interaction, and at the same time, it is not expected that the *ta* size is too large in real space to burden users. Therefore, there is a certain size difference between *ta* and *vp*, and *rh* may reach out beyond the boundary of *ta* and fail to obtain tactile feedback during interaction. In this work, tactile redirection technology is applied to solve the position and tactile inconsistencies caused by the size differences between *ta* and *vp*, and to provide real-time tactile feedback for user interaction in HMD-VR environment, enjoying the reflection of African philosophy in African literature.

Tactile redirection has two prerequisites, which is that the physical target *pt* (the position of *pt* is *P*_*pt*_) and the virtual target *vt* (the position of *vt* is *P*_*vt*_) must be located. *pt* is always in a range of *ta*, whereas *vt* must be selected by the user. Hence, in order to allow users to freely select the redirection target, this work designs a redirection target selection method, which allows users to select the redirection target by staring and hand movements.

Both virtual hand *vh* and physical hand *rh* are initially located at *P*_0_. In the process of *vh* moving to *vt*, the redirection algorithm gradually shifts *vh* toward *rh* and away from the physical target *pt*. Vision plays a dominant role in VR scenes, and the deviation in the moving direction of *vh* will induce the user’s *rh* to move to the *pt* to correct the moving trajectory of *vh*. Finally, when *rh* touches *pt*, *vh* reaches *vt*.

*P*_0_, *P*_*vt*_, and *P*_*pt*_ are required for tactile redirection. Assuming that the spot of virtual scene *P*_*vt*_ should be mapped to the spot of physical scene *P*_*pt*_, the total offset *D* is calculated as follows.


(3) $$DP_{vt}-P_{pt}$$

As *rh* begins to move from initial position *P*_0_ to *vt*, the offset *D* is gradually added to *P*_*vh*_, that is,


(4) $$\delta \left|P_{rh}-P_{0}\right|/\left|P_{rh}-P_{0}\right|\left|P_{rh}-P_{pt}\right|$$

(5) $$P_{vh}=P_{vh}+\delta D$$

where δ is the displacement ratio, which ranges from 0 to 1. When *rh* is at *P*_0_, *rh* is 0. When *rh* reaches *pt*, *rh* is 1.

## Electroencephalogram Emotion Recognition Based on Multiscale Convolution Kernel Convolutional Neural Networks

It is very important for users to recognize their emotions in the immersive experience of African literature. The psychological state of users can be effectively judged through emotion recognition to push appropriate content to users, so that users can better understand the reflection of African philosophical thoughts in African literature. The EEG signal is generated from the neural activity of the cerebral cortex. The cerebral cortex signal recorded in a non-invasive way that can reflect the activity state of the brain to a certain extent. Emotion recognition using EEG signals has become a mainstream research method. The EEG sensors are widely used to detect EEG signals and analyze the control intention of the human brain.

Anger, disgust, fear, happiness, sadness, surprise, and neutrality are the seven states that make up human emotions. In general, the bioelectricity of head collected by EEG sensor includes EEG and electromyography (EMG), and EMG contains abundant information related to emotion. Medical research shows that when people have these seven emotions, the activity of two muscles in the frontal region and two muscles in the central region of the head increases significantly. These four channels of EEG sensors produce a large amount of EEG signals generated by facial movements. The above-mentioned four channels contain rich facial action information. After the original collected signals are preprocessed by clipping, smoothing, and normalization, the standard EEG information flow is formed and sent to the CNN for emotion recognition.

### An Improved Multiscale Convolution Kernel Convolutional Neural Networks

The traditional artificial neural network consists of input layer, hidden layer, and output layer. Based on the CNN, the hidden layer is the full connection layer, and convolution layer and pooling layer are added between the input layer and the full connection layer. Through multilayer convolution, more abstract signal features are continuously extracted to enhance the effective signal features and weaken the noise signal features (Selvaraju et al., 2020). In general, the convolution layer of CNN adopts a single-size convolution kernel. To extract the deeper features of signals, multiple convolution layers need to be constructed, which complicates the network structure. The increasing of network layers will multiply the network parameters, which is not conducive to the rapid convergence of the network and seriously affects the network performance. Therefore, based on the classic CNN model, this work improves the convolutional layer of CNN by adding convolution kernel of different scales, which expands the dimension of feature extraction of the convolutional layer and reduces the number of convolutional layers. Meanwhile, the complexity of the network is also reduced, and the network performance is greatly improved. Multiscale convolution kernel CNN model can set multiple convolution kernels of different sizes in convolution layer and extract features of different dimensions of data at the same time. In multiscale convolution kernels, the small perception field of convolution kernels is also small, and so the detection of detail features is relatively good. Large scale convolution kernel takes into account the information of large perceptual fields, so it can ignore a large amount of noise and detect the overall features more accurately.

The multiscale convolution kernel CNN model designed in this work has five layers. The first layer is the input layer, and the EEG signals are cut into *M* × *N* × 1 size as the input of the multiscale convolution kernel CNN model. The second layer is the convolution layer. Multiscale convolution is used to check the input signals for feature extraction of different dimensions. The dimensions of multiscale convolution kernels are set as *M* × 5 × 1, *M* × 3 × 1, and *M* × 1 × 1, 128 convolution kernels for each size. The third layer is the pooling layer, which adopts the spatial pyramid pooling (SPP). The fourth layer is the full connection layer, which prepares the data for classification. The fifth layer is the output layer, which is use Softmax classifier to achieve three classifications.

### Methodology

#### Loss Function

The multiscale convolution kernel CNN outputs the convolution between the learnable convolution kernel and the convolution of this input layer through forward propagation as the input of the next layer, and revises the network weights and biases of each layer through the back propagation of errors. The forward propagation of multiscale convolution kernel CNN is defined as follows.


(6) $$y_{n}^{p}=f\left(\sum_{m\in B_{n}}x_{m}^{p-1}*l_{mn}^{p}+b_{n}^{p}\right)$$

where $x_{m}^{p-1}$ is the input signal of the *m*th feature map of *p*-1 layer. $y_{n}^{p}$ is the output value of the *n*th feature map of the layer *p*. *B_n_* is a group of feature map. The symbol * stands for convolution. The term $l_{mn}^{p}$ is the learnable convolution kernel between the *m*th feature map of layer *p*-1 and the *n*th feature map of layer *p*. In the equation, *b* is the offset of the output feature map. Rectified linear unit (ReLU) activation function is adopted in this network architecture. The loss function is defined as follows.


(7) $$\begin{array}{c} LF=\sum_{m}\sum_{ny_{m}}\left\{max\left[0,f{\left(x_{m};w\right)}_{n}-f{\left(x_{m};w\right)}_{y_{m}}+TE\right]\right\}\\ \;+\gamma \sum_{l}\sum_{p}w_{l,p}^{2} \end{array}$$

where *x*_*m*_ is the input, *n* is the prediction result of a single sample, and *y*_*m*_ is the result of a real category, *w* is the weight parameter, and *f*(⋅) is the output activation function. The term $\gamma \sum_{l}\sum_{p}w_{l,p}^{2}$ is the regularization penalty term, and γ is the penalty coefficient. *l* and *p* are the row and column of the weight parameters, respectively.

#### Softmax Classification

As we can recollect, in case of a binary classification problem, the sigmoid function can tell us which class the output belongs to. But when there are multiple classes or classed greater than two, that is where softmax classification comes in handy. Softmax classification, also known as normalized probability distribution, presents the results of multiple classes in probabilistic terms, and it can be defined as follows.


(8) $$s\left(a_{i}\right)=e^{a_{i}}/\sum e^{a_{j}}$$

where *j* is the number of classes, *a_i_* represents the linear prediction probability of the *i*th class, and *a_j_* is the sum of the linear prediction probability of *i* classes. Here *s*(*a*_*i*_) represents the normalized prediction result of each class. Adam gradient algorithm is used for back propagation.

#### Spatial Pyramid Pooling

The convolutional layer of CNN can process a number of EEG signals, whereas the feature number of the full connection layer is fixed. Therefore, the input size of the full connection layer needs to be fixed when the network input is made. The SPP (Yu et al., 2020) can transform feature maps of arbitrary size into feature vectors of fixed size.

## Experiment and Results Analysis

### Setup

**FIGURE 2:**
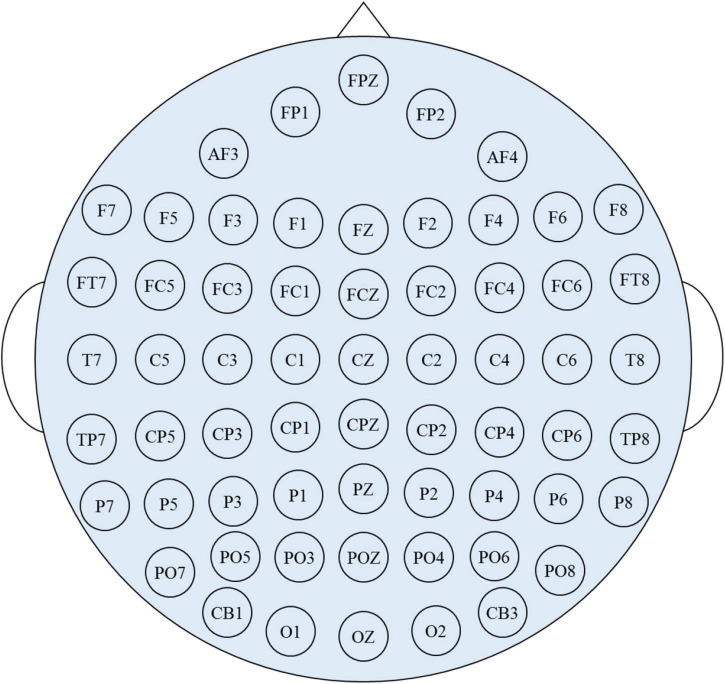
The 62-channel EEG signals.

The simulation is running on a computer with Intel i9-10850K, CPU 3.6 GHz, 32GB RAM 3333 MHz, GPU NVIDIA RTX 3070 Ti 8 GB, Unity3D engine. Arpara VR 5K Headsets are used for HMD-VR device. Leap motion is used as a depth camera for tracking hands and tablet-aid. The size of the tablet-aid is 20 cm × 20 cm, and the corresponding virtual plane is 30 cm × 30 cm. Navigation (NAV), selection (SEL), and manipulation (MA) functions are selected as validation for interaction. The VR scene is taken Things Fall Apart, the masterpiece by famous Nigerian novelist Chinua Achebe. To test the interactive performance of the interactive system in this paper, 20 participants are invited to test. Some of them are not familiar with VR interaction environment, and some understand it, but definitely, they are very familiar with the interaction mode of smart tablet devices. Table 1 shows the network parameters of EEG emotion recognition based on multiscale convolution kernel CNN. The multiscale convolution kernel CNN uses SEED dataset for simulation, which includes 62 channel EEG signals and shows in Figure 2.

**TABLE 1:** Parameters setting.

Parameter	Setting
Batch size	64
Activation function	ReLU
Classifier	Softmax
Number of full connection layer	2
Pooling	Spatial pyramid pooling
Optimizer	Adam
Learning rate	0.002

### Comparison Analysis of Human–Computer Interaction in Head-Mounted Display-Virtual Reality Environment Based on Position Tracking and Tactile Redirection

#### Consistency Verification of the Interaction System Proposed in This Work and Smart Tablet

The interaction system proposed in this work provides real-time tactile feedback for interaction with tablet-aid, and the interaction mode between users and the virtual plane can be completely consistent with the operation mode of smart tablet. To verify the consistency between the interaction system proposed in this work and the interaction of smart tablet, a comparative experiment is conducted with real smart tablet. In this work, the experimental scene is completely transplanted to the smart tablet device (Lenovo Pad Pro, Android 11), and the same interaction system is also designed for the Pad. In this experiment, 20 participants are first asked to complete appreciation and record relevant data in the HMD-VR environment using the interactive system in this work. After a proper rest, 20 participants are asked to directly use smart tablet to complete the same appreciation and record relevant data in real scenes.

**FIGURE 3:**
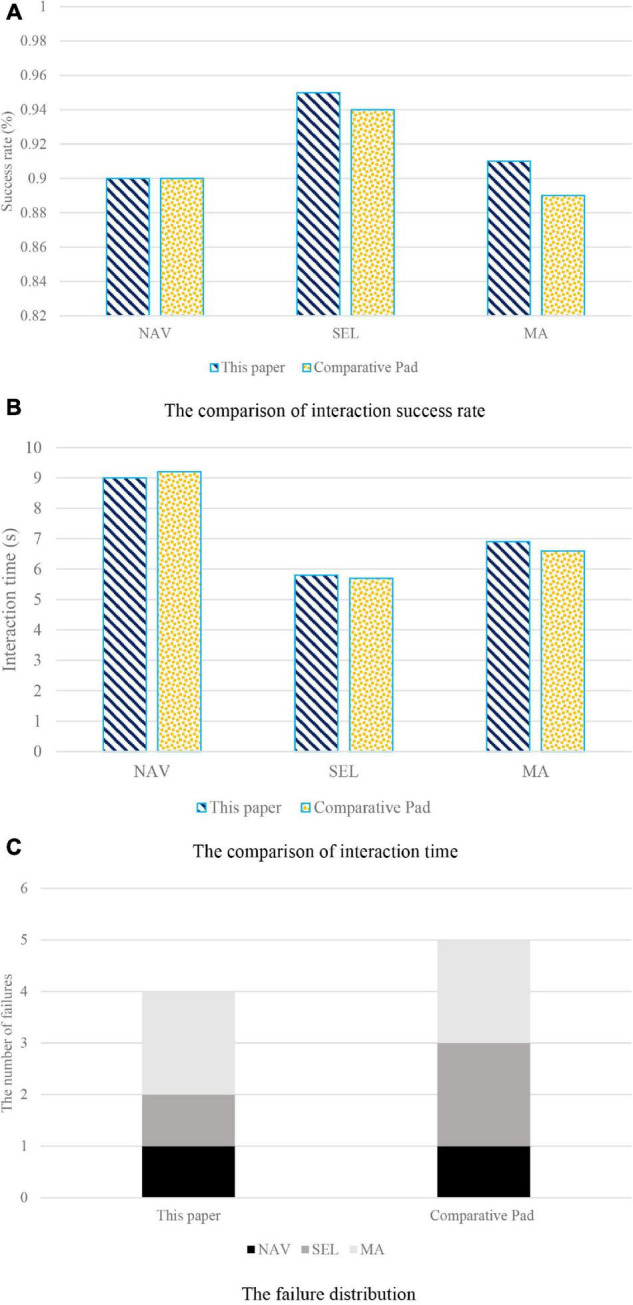
Consistency verification of the interaction system. **(A)** The comparison of interaction success rate. **(B)** The comparison of interaction time. **(C)** The failure distribution.

As can be seen from Figure 3, in terms of the success rate of interaction, the proposed method in this work and comparative Pad have high success rate of interaction. The interaction failures of proposed method in this work and comparative Pad are similar in number and mainly focus on the SEL and MA process with complicated operation steps. Since the proposed method in this work and comparative Pad share the same interaction paradigm, there is a similar distribution of failures. The proposed method in this work and comparative Pad are close to each other in the time of NAV, SEL, and MA. It can be seen that the tactile redirection method in this work has good support for the continuous interaction of adjacent objects, making the interaction system proposed in this work closer to the interaction efficiency of the smart tablet. Therefore, the interactive system proposed in this work is consistent with the smart tablet in the performance of completing interactive appreciation.

#### Consistency Verification of the Interaction System Proposed in This Paper and Other Interaction System

To test the specific interaction performance of the interactive system proposed in this work in the scene of African literature and African philosophy appreciation, this work also conducts comparative experiments with other two interactive systems, which are HTC VIVE PRO (HTC) and VALVE INDEX 2.0 STEAM (VALVE). In the HTC interactive system, navigation is carried out in point-to-point leaping, target selection is carried out by ray, and manipulation is carried out by close contact grabbing. In VALVE interactive system, the controller handles navigation and manipulation. After the staring time reaches a threshold, the selection operation is completed, and the system is controlled by the buttons on the controller. In the comparative experiment, 20 participants complete the appreciation of African literature and African philosophy using the above two interactive systems and record relevant data. To prevent fatigue accumulation, participants use an interaction system to complete the appreciation and then rest for 5 min. The interaction success rate, interaction time, and failure distribution of the three interaction systems are calculated.

**FIGURE 4:**
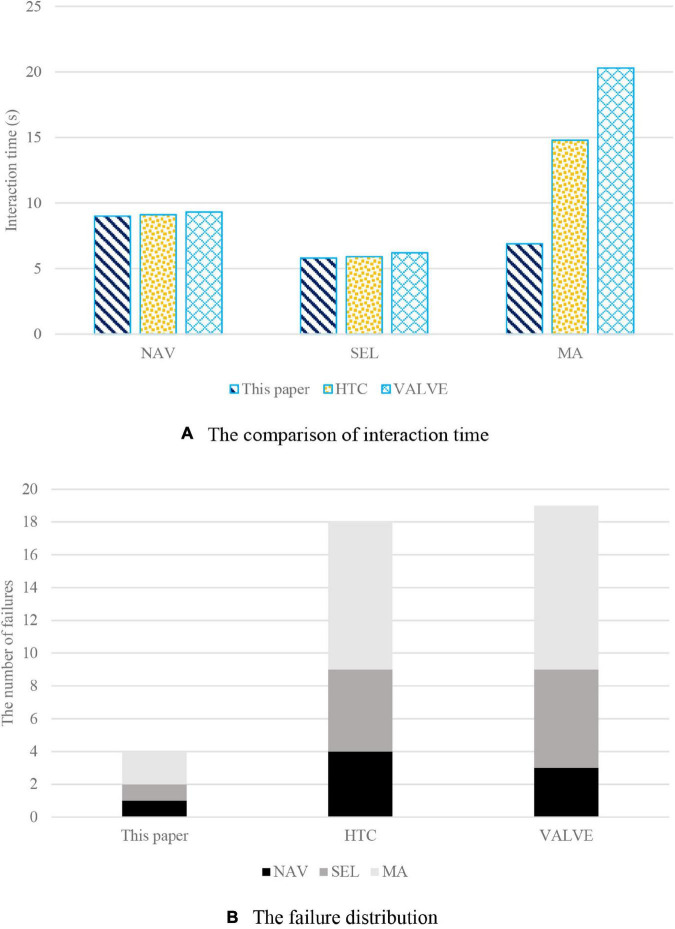
The comparison of interactive system. **(A)** The comparison of interaction time. **(B)** The failure distribution.

As can be seen from Figure 4A, there is no significant difference in the interaction time between the proposed method in this work, HTC, and VALVE, and the interaction time is about 9 s. For the selection function, the interaction time is about 6 s. The interaction time is related to the crosssection size of the target, and the larger the area is, the less time it takes. Therefore, HTC and VALVE are easily affected by the target size, and the VALVE interaction system is most obviously affected. For the proposed method in this work, although the selection time is also affected by the target size to a certain extent, it is not as obvious as HTC and VALVE. In terms of manipulation of African literature and African philosophy appreciation, the proposed method in this work spends less time than HTC in manipulating the target. The proposed method in this work can move and rotate objects conveniently and quickly and complete the manipulation in a short time by introducing the interaction paradigm of smart tablet through tablet-aid. HTC takes longer because of its complexity. On the other hand, VALVE is unable to perform manipulation tasks in African literature and African philosophy appreciation due to its inability to accurately move and manipulate objects.

It is not just the interaction time that is affected by target size, but also the success rate of the interaction. As shown in Figure 4B, the number of interactive failures is counted in this work, among which the number of failures of HTC and VALVE is significantly higher than that of the proposed method in this work. Furthermore, the number of interaction failures is mostly on HTC and VALVE’s manipulation functions. From the perspective of interaction performance, the interaction system in this work introduces the interaction paradigm of smart tablet through tablet-aid, which can better meet users’ interaction needs in the scene of African literature and African philosophy appreciation and provide efficient navigation, selection, and manipulation control. In terms of interactive experience, HTC and valve without plane aid cannot provide tactile feedback for interaction, so the immersion of interaction is not high. While the interactive mode of the proposed method in this work with tablet-aid not only improves the interactive ability, but also greatly improves the immersion of interaction, thanks to real-time tactile feedback.

#### Quality of Experience

The quality of experience (QoE) is the most important in HCI-oriented African literature and African philosophy appreciation. The feelings and experiences of 20 participants on the three interaction modes are investigated through the questionnaires, which are listed as follows.

Q1: Feel dizzy and sick during use.

Q2: Experience a high degree of immersion during use.

Q3: Can meet interaction needs.

Q4: Tired during use.

**FIGURE 5:**
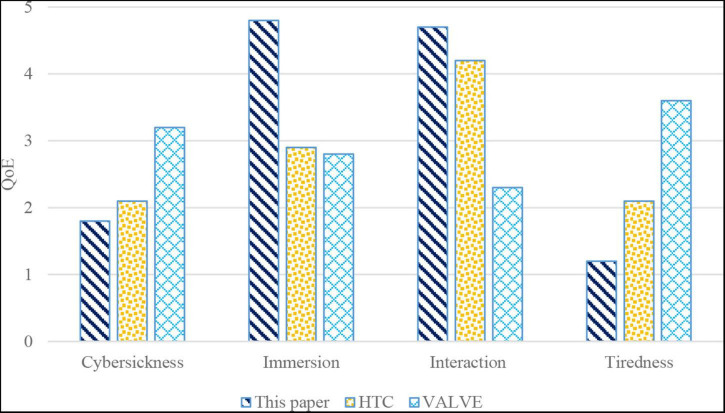
The results of questionnaires.

For the above questions, participants give scores from one to five, with one representing completely disagree and five representing completely agree. The score of each interaction mode is shown in Figure 5.

The statistical results in Figure 5 show that participants generally feel that VALVE caused severe cybersickness. VALVE not only makes participants feel dizzy, but also tired. VALVE has to constantly control the perspective to switch between interactive objectives and hold the viewpoint to complete the interaction. On the other hand, HTC shows no obvious differences in participants’ feelings due to the convenience. However, because the interaction can be easily completed on the virtual plane, the method proposed in this work is in line with participants’ habits of using smart tablet, and there is no obvious feeling of cybersickness and tiredness. In terms of interactive ability, both the method proposed in this work and HTC can meet users’ interactive appreciation. However, it can be found that participants generally believe that the comprehensive interaction ability of VALVE is weak, and in immersion, participants generally rated HTC and VALVE’s interactive experiences as less immersive. Since HTC and VALVE interact without tactile feedback, the interaction is not real and natural. The method proposed in this work has a strong immersion and good interactive experience because it provides real tactile feedback for interaction.

### Comparison Analysis of Electroencephalogram Emotion Recognition Based on Multi-Scale Convolution Kernel Convolutional Neural Networks

#### Channel Selection

In practical applications, it is very important to use fewer EEG channels to achieve high-precision emotion recognition. Therefore, the preprocessed data of SEED dataset is used to explore the impact on brain regions and electrode number on emotion recognition accuracy. As the regions affecting emotions are mainly in the temporal lobe, frontal lobe, and anterior half of the brain (Li et al., 2019), Figure 6 shows the channels under the five conditions selected in this work: (1) 4-channel (T7, T8, TP7, TP8); (2) 6-channel (FT7, FT8, T7, T8, TP7, TP8); (3) 9-channel (FP1, FPZ, FP2, FT7, FT8, T7, T8, TP7, TP8); (4) 15-channel (FP1, FPZ, FP2, FT7, FT8, T7, C5, C6, T8, TP7, CP5, CP6, TP8, P7, P8); and (5) 33-channel (FP1, FPZ, FP2, AF3, AF4, F7, F5, F3, F1, FZ, F2, F4, F6, F8, FT7, FC5, FC3, FC1, FCZ, FC2, FC4, FC6, FT8, T7, C5, C6, T8, TP7, CP5, CP6, TP8, P7, P8).

**FIGURE 6:**
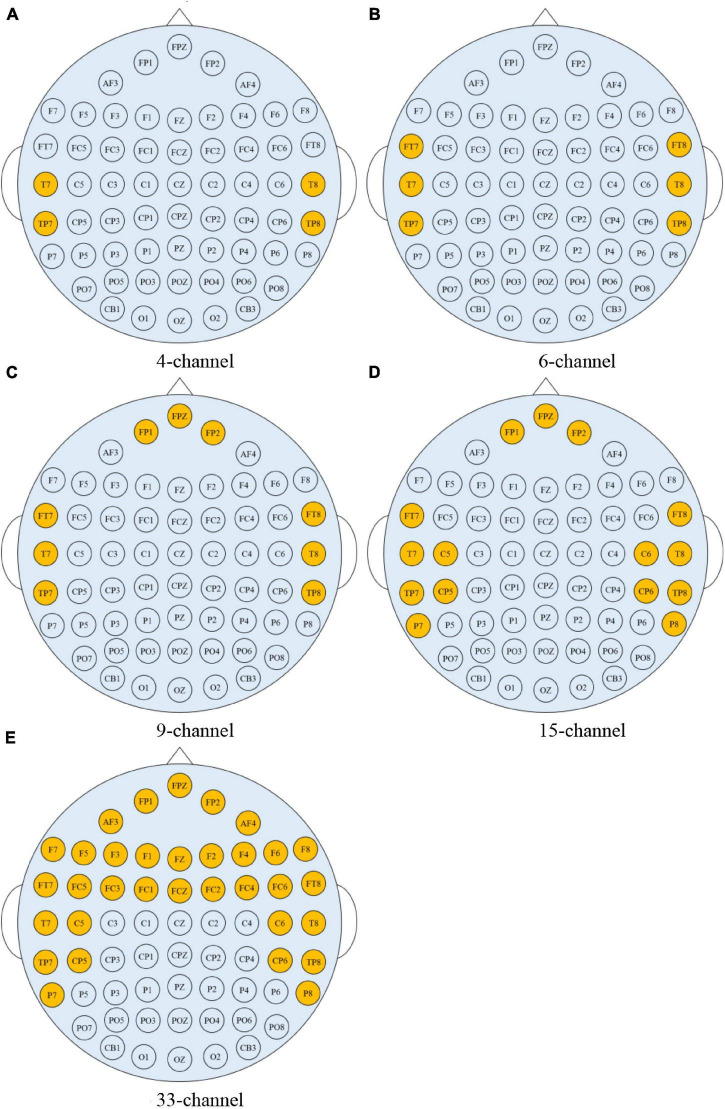
Scalp electrode distribution of five different channels. **(A)** 4-channel. **(B)** 6-channel. **(C)** 9-channel. **(D)** 15-channel. **(E)** 33-channel.

Table 2 shows the classification accuracy of each volunteer in five different channels. The accuracy of 4-channel, 6-channel, 9-channel, 15-channel, and 33-channel are 76.27, 80.62, 81.31, 90.63, and 91.36%, respectively. As can be seen from the accuracy, the classification accuracy increases with the increasing number of channels, and the accuracy of 33-channel is almost close to that of 62-channel, indicating that the 33-channel contains most discriminant information of emotion recognition. In addition, the time for classification using 33-channel data is nearly 80% lower than that using the 62-channel data. Channel selection can effectively remove redundant information, and so this work uses the 33-channel for the simulation of African literature and African philosophy appreciation.

**TABLE 2:** Classification accuracy of each volunteer on 4/6/9/15/33-channel.

Volunteer number	The number of channels					
	4	6	9	15	33	62
1	66.29	68.88	69.96	74.36	84.02	89.35
2	62.71	69.49	71.31	77.19	86.19	90.59
3	70.47	76.54	79.43	84.79	92.80	91.05
4	67.85	71.56	74.38	76.15	85.66	82.60
5	69.15	75.28	77.48	79.21	97.98	88.16
6	82.13	84.56	85.14	84.61	94.64	93.21
7	79.54	82.11	81.26	88.22	88.33	91.43
8	79.01	84.67	85.98	88.56	93.87	93.19
9	76.63	78.92	83.15	84.65	91.21	92.81
10	80.02	85.91	78.26	79.98	89.71	90.98
11	82.14	84.16	88.34	89.39	95.03	93.62
12	67.05	73.64	77.56	81.34	87.72	89.56
13	71.46	79.20	79.98	83.07	92.51	90.45
14	67.56	77.11	78.59	81.65	84.23	82.65
15	78.64	88.64	83.64	92.36	88.69	94.57
16	79.61	89.61	90.16	87.86	94.01	92.40
17	81.07	79.48	79.65	88.21	87.59	95.12
18	86.51	84.12	78.07	93.36	88.00	94.87
19	87.63	86.35	87.87	94.50	95.57	96.01
20	89.98	92.16	95.96	96.53	94.81	94.64
Average accuracy	76.27	80.62	81.31	85.30	90.63	91.36

#### Classification Accuracy

To further verify the superiority of feature extraction and classification of EEG emotion recognition based on multiscale convolution kernel CNN, which will be compared with the feature extraction and classification method using SEED dataset. Three evaluation metrics commonly used in classification are used to compare and evaluate the quality of simulation results, which include precision rate (*P*), recall rate (*R*), and F1 score (F1). *P* is the accuracy of extraction results. *R* is the coverage degree of the extraction results to the correct keywords. F1 is a comprehensive evaluation index of harmonic average of *P* and *R*. The calculation for *P*, *R*, and F1 are defined as follows from Eqs 9–11. The transferable ranking convolutional neural network (TRk-CNN) (Jun et al., 2021), novel intelligent fault diagnosis approach based on principal component analysis (PCA) and deep belief network (DBN) (PCA-DBN) (Zhu et al., 2020), and dual path CNN-recurrent neural network cascade network (DPCRCN) (Yang et al., 2019) are selected for comparison. While TRk-CNN is used to show a high correlation with each other when the classes of images to be classified, PCA-DBN is a novel intelligent fault diagnosis approach, and DPCRCN achieves an end-to-end learning for classification.


(9) $$P\frac{TP}{TPFP}$$

where *TP* is the number of true positives, *FP* is the number of false positives, and *TPFP* means the number of predicted positives.


(10) $$P\frac{TP}{TPFN}$$

where *FN* is the number of false negatives, and *TPFN* is the number of true positives.


(11) $$P\frac{2PR}{PR}$$

**FIGURE 7:**
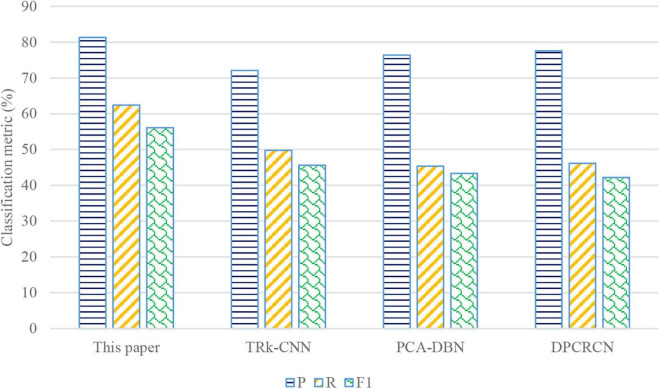
The comparison of classification metric of the four algorithms.

As can be seen from Figure 7, *P*, *R*, and F1 of the algorithm proposed in this work are all higher than other three baselines. The gradient disappearance will not come out in ReLU activation function, which increases the non-linear relationship between layers of neural network. At the same time, ReLU activation function makes the output of some neurons become zero, which results in the sparsity of the network, reduces the interdependence of parameters, alleviates the over-fitting problem, and improves the accuracy of classification. Moreover, the introduced SPP is independent of the specific CNN network architecture, which enhances the *R* and F1 of the multiscale convolution kernel CNN.

#### Classification Time

**FIGURE 8:**
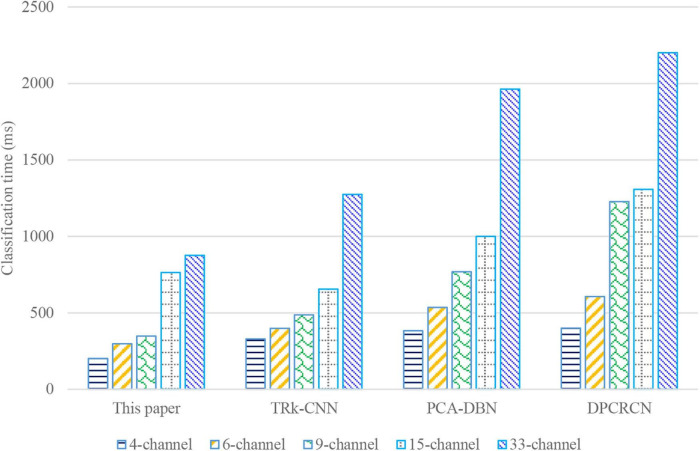
The comparison of classification time of the four algorithms.

As can be seen from Figure 8, the classification time of the proposed algorithm in this work is significantly better than that of the other three baselines. The classification time of the proposed algorithm for 15-channel is similar to that for 33-channel, while the classification time of 33-channel for the other three baselines is almost twice that of the 15-channel. This also explains the rationality of choosing 33-channel for simulation as the appreciation African literature and African philosophy in this work.

## Conclusion

As a way to kind of further the process of appreciating the reflection of African philosophy in African literature, this work proposes HCI-oriented appreciation method. The interaction system proposed in this work ensures high interaction efficiency, improves users’ immersion and interactive experience, and solves the interaction problems in appreciation well. The tactile redirection is improved to enhance the flexibility and efficiency of interaction. Furthermore, this work takes emotional EEG signals as feature extraction objects and improves them on the basis of classical CNN model. Multiscale convolution kernel CNN is proposed for feature extraction and classification of emotional EEG, and its effectiveness is verified on SEED dataset. The experimental results demonstrate that the method proposed in this work has a good performance in terms of immersion and interaction of African literature and African philosophy appreciation.

The method proposed in this work performs well in VR appreciation scene interaction, and the interaction efficiency and interactive experience will not be affected by shield, but the selection method of redirection target is not natural enough. In the future, it is hoped that users’ interaction intention can be accurately predicted by detecting the movement track of their hands, so as to further improve users’ interactive experience. At the same time, electrooculogram signals and EEG signals are merged to design a multimode CNN for emotion recognition.

In particular, to make the readers more easily follow this work, the commonly used abbreviations are listed in Table 3.

**TABLE 3:** Abbreviations in alphabetical order.

Abbreviation	Full name
AR	Augmented Reality
CNN	Convolutional Neural Networks
DPCRCN	Dual Path CNN-Recurrent Neural Network Cascade Network
EEG	Electroencephalogram
EMG	Electromyography
F1	F1 score
HCI	Human-Computer Interface
HMD	Head-Mounted Display
HMD-VR	Head-Mounted Display-Virtual Reality
MA	Manipulation
NAV	Navigation
pt	Physical target
P	Precision rate
PCA-DBN	Principal Component Analysis and Deep Belief Network
P_pt_	The position of pt
P_rh_	The position of rh
P_ta_	The position of ta
P_vh_	The position of vh
P_vp_	The position of vp
P_vt_	The position of vt
QoE	Quality of Experience
R	Recall rate
ReLU	Rectified Linear Unit
rh	Right hand
SEL	Selection
SPP	Spatial Pyramid Pooling
ta	Tablet-aid
TRk-CNN	Transferable Ranking Convolutional Neural Network
vh	Virtual hand
vp	Virtual plane
VR	Virtual Reality
vt	Virtual target

## Data Availability Statement

The raw data supporting the conclusions of this article will be made available by the authors, without undue reservation.

## Author Contributions

JW contributed to the writing and methods. YP contributed to the material collection and project support.

## Conflict of Interest

The authors declare that the research was conducted in the absence of any commercial or financial relationships that could be construed as a potential conflict of interest.

## Publisher’s Note

All claims expressed in this article are solely those of the authors and do not necessarily represent those of their affiliated organizations, or those of the publisher, the editors and the reviewers. Any product that may be evaluated in this article, or claim that may be made by its manufacturer, is not guaranteed or endorsed by the publisher.
